# Embolization for type 2 endoleak with sac expansion after endovascular repair of abdominal aortic aneurysm: safety and effectiveness

**DOI:** 10.1186/s40064-016-1934-x

**Published:** 2016-03-02

**Authors:** Kenji Kajiwara, Takuji Yamagami, Masaki Urashima, Hideki Tomiyoshi, Hideaki Kakizawa, Rika Yoshimatsu, Masaki Ishikawa, Kazuo Awai

**Affiliations:** Diagnostic Radiology, Graduate School of Biomedical Sciences, Hiroshima University, 1-2-3 Kasumi, Minami-ku, Hiroshima, 734-8551 Japan; Diagnostic Radiology, Hiroshima City Hospital, 7-33 Motomachi, Naka-ku, Hiroshima, 730-8518 Japan; Radiology, Higashihiroshima Medical Center, 513 Saijyou-tyo, Hiroshima, 739-0041 Japan; Radiology, Hiroshima Red Cross Hospital and Atomic-Bomb Survivors Hospital, 1-9-6, Senda-machi, Naka-ku, Hiroshima, 730-8619 Japan

**Keywords:** Vascular, Interventional, Embolization, Endovascular aneurysm repair, Type 2 endoleak

## Abstract

To evaluate the safety and outcome of embolization as treatment for persistent type 2 endoleak (T2EL) occurring after abdominal aortic stent graft implantation. This retrospective study included seven consecutive patients (one female, six males, mean age 72 years, range 66–88 years) with T2EL between January 2011 and September 2012. In all, T2EL was associated with an increase more than 5 mm in the aneurysm. The endoleak cavity or feeding artery was embolized with coils and/or *n*-butyl cyanoacrylate. Clinical success was defined as regression or stabilization of the aneurysm sac irrespective of residual endoleaks on follow-up CT studies. At the time of T2EL intervention, mean aneurysm sac diameter was 63 mm (range 52–72 mm), and mean increase size of aneurysm sac diameter was 7 mm (range 5–13). Mean follow-up period was 6.0 ± 6.2 months (range 3–18 months). Our technical success rate was 100 %. Clinical success was obtained in 5 (71.4 %) of the seven patients. One patient was embolized three times due to sac expansion. T2EL was treated by transarterial embolization in eight procedures, and one procedure was performed by direct puncture embolization. There were no major complications; two procedures elicited minor complications: transient back pain and muscle weakness of the left lower leg. We suggest embolization was safe and effective treatment, a less invasive treatment option comparison to open repair, as one choice to address T2EL.

## Background

Over the last several years, endovascular aneurysm repair (EVAR) has become an effective alternative to open surgery for abdominal aortic aneurysms (AAA). Endoleaks are known as the most common complications of EVAR and are classified into four types according to cause. Type 1 endoleak is caused by antegrade flow into the aneurysm sac, which may occur at the attachment site. Type 2 endoleak (T2EL) is retrograde flow in the collateral side-branch arteries, type 3 endoleak is antegrade flow at the junction point between graft components, and type 4 endoleak is caused graft wall porosity (Rhee et al. 2003; Haulon et al. 2001; Faries et al. 2003; Axelrod et al. 2004). T2EL is the most frequently occurring endoleak and occurs in approximately 10–25 % of patients who undergo EVAR (Rhee et al. 2003; Baum et al. 2002; Stavropoulos et al. 2005). Blood enters the aneurysm through a circuitous route, emptying into the aneurysm sac via retrograde flow. This flow is usually through one or more lumbar arteries or the inferior mesenteric artery (Rhee et al. 2003; Baum et al. 2002; Mansueto et al. 2005).

There is widespread agreement that type 1 and type 3 endoleaks require urgent treatment (Rhee et al. 2003), but no such consensus exists at this time regarding treatment of T2ELs. In fact, persistent T2ELs would often decrease or disappear without therapy (Funaki et al. 2012; Nevala et al. 2010). However, they are sometimes associated with expansion and rupture of the aneurysm sac, then a treatment is required. Treatment options for T2EL include transarterial embolization (TAE), embolization after direct puncture (DP) by the abdominal or translumbar approach, transcaval embolization, and open surgical repair (Baum et al. 2002; Stavropoulos et al. 2005; Mansueto et al. 2005; Nevala et al. 2010; Ellis et al. 2003). The embolic materials used in embolization have been coils, *n*-butyl-2-cyanoacrylate (NBCA) (Histoacryl; B-Braun, Germany), thrombin, ethylene vinyl alcohol copolymer, and gelfoam (Baum et al. 2002; Stavropoulos et al. 2005; Nevala et al. 2010; Ellis et al. 2003; Khaja et al. 2014).

The purpose of the present study was to evaluate the usefulness and safety of endovascular treatment for T2EL.

## Methods

### Patients

Written informed consent was obtained from all patients. Between January 2011 and September 2012, seven consecutive patients (six men and one woman, mean age 72 years, range 66–88 years) underwent nine sessions of embolization for T2EL at affiliated hospitals. All institutions do not require institutional review board approval for this type of clinical observational study, and our study carried out according to all affiliated hospitals guidelines. In all patients, T2EL was detected on follow-up computed tomography (CT) scans and an increase in the size of the aneurysm was proven. Sac size was measured at the largest minor axis diameter on the axial image. Indication for treatment of T2EL was defined as an increase in the aneurysm >5 mm in diameter compared with CT findings performed immediately after the EVAR procedure regardless of the length of the T2EL. Clinical symptoms were not present in any patient. Candidates for embolization were selected by consensus between interventional radiologists and cardiovascular surgeons at a case presentation conference. At the time of the T2EL interventions, the mean aneurysm sac diameter was 63 mm (range 52–72 mm) and mean increase in the diameter of the aneurysm sac was 7 mm (range 5–13 mm). Average time between primary EVAR and the procedure to treat T2EL was 20 months (range 13–50 months). The types of endograft used were the Zenith (Cook, Bloomington, IN, USA) in four patients and the EXCLUDER (Gore, Flagstaff, AZ, USA) in three patients.

**Fig. 1:**
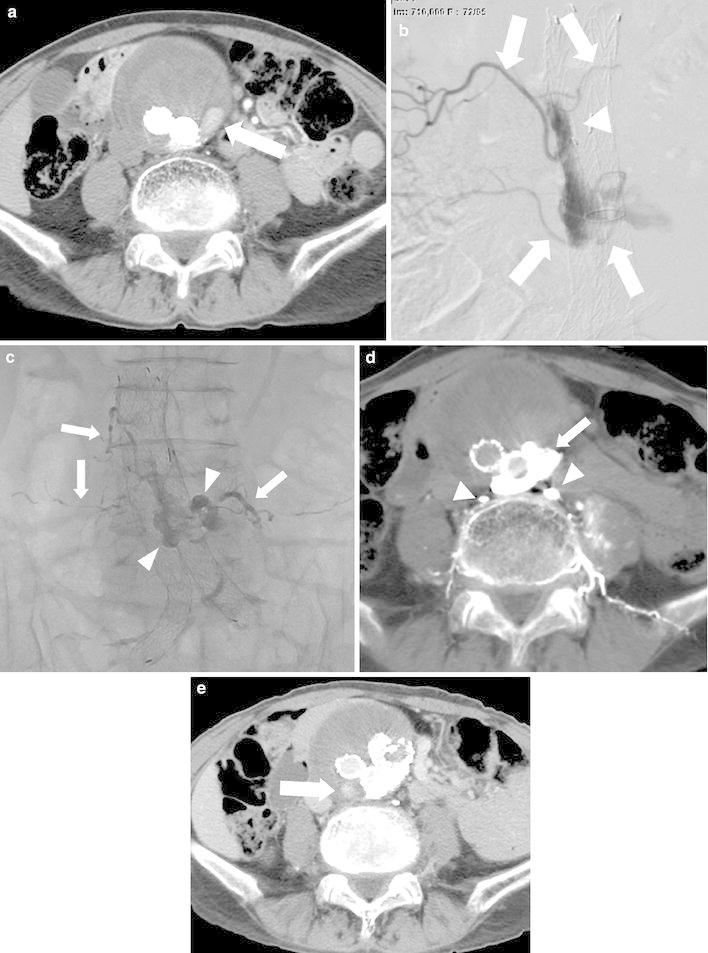
Images of an 88-year-old man with T2EL (case no 1, Table 1). **a** Enhanced CT showed that the aneurysm expanded with persistence of T2EL (*arrow*) 15 months after EVAR. **b** Endoleak angiogram performed via a microcatheter demonstrated that the endoleak involved four vessels (*arrows*). *Arrowhead* indicates the micro catheter tip that was advanced into the endoleak sac through the left fourth lumbar artery. **c** Roentgenogram showed NBCA injected to fill simultaneously the aneurysmal sac (*arrowheads*) and the involved lumbar arteries (*arrows*). **d** Plain CT showed radiopaque NBCA accumulations in the endoleak sac (*arrow*) and lumbar arteries (*arrowheads*). **e** Enhanced CT obtained 6 months after the embolization showed another small endoleak (*arrow*) with no further expansion of the aneurysm

The embolization technique involved entering the endoleak cavity and/or feeding artery with a mixture of NBCA and lipiodol and/or coils by TAE or DP. To perform TAE, the common femoral artery was punctured and a 4-F catheter was inserted. Then a microcatheter was coaxially advanced. Catheterized arteries were the ascending lumbar arteries into which the catheter was advanced via the hypogastric artery or the inferior mesenteric artery into which the catheter was advanced via the superior mesenteric artery through the middle ascending left colic arcade or arc of Riolan. If possible, the microcather was further advanced to the portion of the endoleak within the aneurysm sac. After an arteriogram from the microcatheter was obtained to decide the point from which embolic agents were inserted, embolization was performed (Fig. 1e). As embolic agents, microcoils and/or NBCA were used. NBCA was used only in patients in whom the sac could be catheterized. The ratio of NBCA to lipiodol was 1:2–10. In patients in whom the sac could not be catheterized (n = 3), the feeding artery was embolized with microcoils as closely as possible to the sac.

**Fig. 2:**
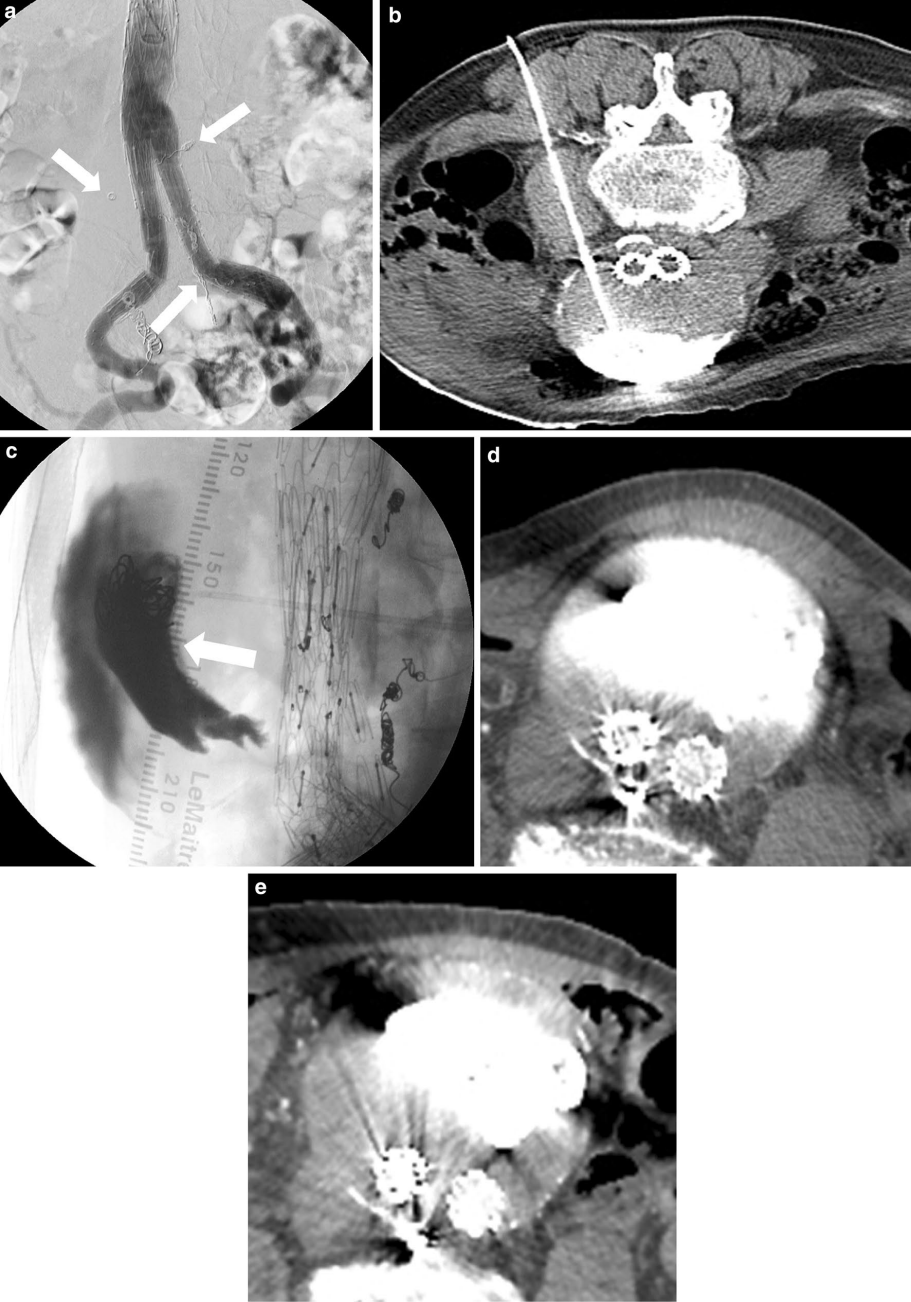
Images of an 87-year-old man with T2EL (case no. 6, Table 1). **a** Angiography in the prone position showed no endoleak before the third embolization. Lumbar artery and middle sacral artery were filled with coils (*arrow*) as a result of previous TAE. **b** Plain CT showed that the tip of the sheath that had been advanced after direct puncture under fluoroscopic guidance was correctly located. **c** Roentgenogram obtained after the third embolization showed NBCA and coils (*arrow*) in the endoleak sac. **d** Enhanced CT obtained 5 days after the third embolization showed NBCA and contrast media in the endoleak sac. Diameter of the aneurysm was 80 mm. **e** Enhanced CT obtained 6 months after the third embolization showed no endoleak; however, growth of the aneurysm sac was confirmed. Diameter of the aneurysm was 83 mm

To perform DP embolization (Fig. 2b), while the patient was in the prone position, the endoleak cavity was punctured with an 18-gauge needle under fluoroscopic guidance. After confirming that blood was aspirated, the 0.035-in. guide wire included in the commercially available kit was advanced into the leak, followed by advancement of a 4-F sheath. Subsequently, the patient was moved from the angiography room to the CT room, in which CT was performed to confirm that the tip of the sheath was correctly located. After the patient returned to the angiography room, a 4-F catheter and microcatheter were inserted into the endoleak cavity. NBCA and coils were used in an attempt to fill the aneurysm sac. The ratio of NBCA to lipiodol was 1:3.

The standard post-embolization surveillance protocol included CT angiography at 3, 6, and 12 months and semi-annually thereafter.

The following parameters were retrospectively investigated: (1) technical success, (2) clinical success, (3) complications, and (4) effectiveness of the treatment. Technical success was defined as occlusion of the endoleak sac or feeding artery confirmed by angiography performed immediately after TAE. With DP, technical success was defined as confirmation by CT performed immediately after embolization that the endoleak was filled with NBCA. Clinical success was defined as regression or stabilization of the aneurysm sac (i.e. shrinkage or no further growth) irrespective of the existence of residual endoleaks during continued follow-up (minimum 3 months follow-up). Procedural and post-procedural complications were recorded according to the Society of Interventional Radiology clinical practice guidelines.

## Results

Overall, nine procedures (eight TAE, 1 DP) were performed for seven patients (Table 1). Embolic agents used were microcoils (n = 6) and NBCA (n = 5). Technical success was achieved in all nine procedures. Clinical success was achieved in five of the seven patients irrespective of technical success. The mean follow-up period for the seven patients was 6 ± 6.2 months (range 3–18 months). There was no instance of aneurysm rupture or aneurysm-related mortality.

**Table 1:** Demographics and results in patients who underwent endoleak embolization

Case	Age (years)/sex	Diameter of aneurysm (mm)	Procedure	Embolization site	Embolic materials	Technical success	Complication	Clinical success	Residual T2El	Size of aneurysm
1	88/M	66	TAE	Endoleak sac	NBCA (1:10)^a^	Yes	Back pain Muscle weekness	Yes	+	No change
2	66/M	57	TAE	Endoleak sac	NBCA (1:10)^a^	Yes	Back pain	Yes	−	No change
3	83/M	72	TAE	Endoleak sac	NBCA (1:10)^a^	Yes	−	Yes	+	No change
4	72/M	60	TAE	Main feeder	Coil	Yes	−	Yes	+	No change
5	66/F	52	TAE	Endoleak sac	Coil + NBCA (1:2)^a^	Yes	−	Yes	+	No change
6	87/M	➀ 72	TAE	Main feeder	Coil	Yes	−	No	+	Increase
		➁ 75	TAE	Main feeder	Coil	Yes	−		+	Increase
		➂ 80	DP	Endoleak sac	Coil + NBCA (1:3)^a^	Yes	−		−	Increase
7	72/M	63	TAE	Main feeder	Coil	Yes	−	No	−	Increase

*TAE* transarterial embolization, *DP* embolization after direct puncture, *NBCA* *n*-butyl-2-cyanoacrylate

^a^The ratio of NBCA and lipiodol

Residual endoleak was recognized in five patients after the first procedure, with the size of the aneurysm sac being unchanged in four patients but increasing in the remaining patient. In that patient, two additional interventions were performed. However, although the endoleak was resolved after the third intervention, enlargement of the aneurysm could not be stopped. Currently the patient cannot receive further treatment because of severe heart failure.

The T2EL disappeared in two of the seven patients after the first embolization. However, an increase in the aneurysm sac was recognized in one of these two patients. In this patient the aneurysm was stable 6 months later, but had increased 5 mm a year after that. The residual endoleak was not visualized on CT angiography. For this patient superior mesenteric and internal iliac angiography was performed, but the endoleak could not be visualized. Because of the continued sac expansion without the presence of endoleak on angiography and CT angiography, this patient is presently being meticulously observed.

There were two minor procedure-related complications. Two patients experienced back pain, one of which developed transient slight muscle weakness of the left lower leg. Both complications were self-limiting and resolved without therapy.

## Discussion

T2EL is the most common complication of EVAR. Previously reported series of T2EL embolization included patients with not only enlarging but also stable aneurysms (Baum et al. 2002; Stavropoulos et al. 2005, 2009; Nevala et al. 2010; Kasirajan et al. 2003). Explanation of the necessity of embolization of a stable aneurysm is based on the existence of near-systemic pressure in the aneurysm sac of patients with T2ELs (Rhee et al. 2003). However, many physicians have the different opinion that treatment for T2ELs is required only when the aneurysm is expanding (Faries et al. 2003; Funaki et al. 2012; Ellis et al. 2003; Steinmetz et al. 2004; Gunasekaran et al. 2013). Many T2ELs will resolve spontaneously and many others that persist do not lead to adverse outcomes. Furthermore, Gallagher et al. (2012) reported that persistent T2ELs without sac expansion were not at risk of rupture. We believe that treating a T2EL without sac expansion has little clinical significance. Hence, we performed embolization of a T2EL for only cases in which sac enlargement was confirmed.

In the present study, clinical success was achieved in five of the seven patients. In one of the two patients experiencing clinical failure, the T2EL disappeared after the third intervention; however, we could not stop enlargement of the aneurysm. In both of these patients with sac expansion even after the final treatment for T2EL, the T2EL was no longer evident on CT angiography, and in one disappearance of the T2EL was also confirmed by angiography. There are two possible reasons for continuous enlargement of the aneurysm. First, T2ELs that are invisible by both angiography and CT angiography exist. Second, there are causes of enlargement of aneurysms other than T2EL. Aziz et al. (2012) reported that percutaneous endovascular intervention for a T2EL with aneurysm sac growth does not alter the rate of aneurysm sac growth. They reported that in 42 patients who underwent a T2EL intervention the mean diameters of the aneurysm were 6.1 ± 1.6 cm at EVAR, 6.6 ± 1.5 cm at the initial T2EL treatment, and 6.9 cm ± 1.7 cm at the last follow-up. There were no significant differences in sac growth pre- and post-T2EL treatment (Aziz et al. 2012). Indeed, there were cases in which the increase in the aneurysm was not arrested after T2EL treatment. On the other hand, in many cases the enlargement was arrested by percutaneous endovascular intervention. Funaki et al. (2012) reported treatment of a T2EL in 16 patients with growing aneurysms, with technical success observed in 88 % (14 of 16 patients) and clinical success achieved in 100 % (16 or 16 patients). Furthermore, with regard to the clinical success rate of treatment of a T2EL with an enlarging aneurysm, Gallagher et al. (2012) reported a rate of 75.9 % (22 of 29 patients) and Steinmentz et al. (2004) reported a rate of 100 % (five of five patients). Hence, we believe that it is worth trying embolization initially and surgical therapy is necessary if embolization fails and the increase of aneurysms continues.

Sac embolization and branch vessel embolization have been the most frequently used procedures for T2EL treatment. Baum et al. (2002) in a comparison of embolization after DP with TAE of only a single feeding artery observed a residual T2EL in 80 % of the TAE group but in only 8 % of the DP group. However, they did not describe the outcome of sac expansion after embolization. In the present study sac embolization was not achieved in any case. In a patient for whom branch vessel embolization was performed, aneurysm growth was arrested (case no. 4, Table 1). On the other hand, in a patient for whom sac embolization was performed, aneurysm growth was not arrested (case no. 6, Table 1). Among five patients with no further growth in the aneurysm, T2EL persisted in four patients at follow-up CT angiography. We believe that in a patient with a growing aneurysm arresting growth and preventing rupture are the best indicators of clinical success, not the presence or absence of an endoleak as shown on CT. Funaki et al. (2012) noted that persistent endoleak was observed after embolization in two patients in whom there was technical failure. But the aneurysm growth was arrested and both patients had maintained stable aneurysms at their follow-up. From this, we believe that decreasing perfusion to the sac may stabilize growing aneurysms in patients with T2EL and the inability to completely eradicate a small endoleak does not necessarily equate clinical failure.

There were two complications in this series: back pain and muscle weakness of the left lower leg caused by non-target branch embolization by NBCA. In both cases, a low viscosity mixture of NBCA and iodized oil (i.e. ratio of NBCA to lipiodol 1:10) was used. Thus, from our limited experience we speculate that the appropriate viscosity might be higher, for example, 1:2–1:3 ratios. Prior coil placement could decrease blood flow in the endoleak sac, which may help to prevent distal embolization by NBCA.

Our study has some limitations. The sample size was small and its design was retrospective and observational. Recurrence has been observed years after embolization. Our follow-up period was not long (mean, 6 months), and the timing of follow-up studies was irregular. Furthermore, there remains a possibility that some of the aneurysms that increased in size for which embolization was performed might have stabilized without any therapy. However, we believe that the high frequency of technical and clinical success and low rate of complications shown in the present study suggest the effectiveness and safety of embolization for T2EL.

In conclusion, embolization of T2ELs effectively arrests growth, as sacs stabilized in many patients with T2ELs after embolotherapy. We suggest embolization might be safe and effective treatment, a less invasive treatment option, as the first choice to address T2EL.
